# Bioprosthetic heart valve degeneration: new insights into structural remodeling and calcification

**DOI:** 10.3389/fcvm.2026.1941295

**Published:** 2026-09-10

**Authors:** Sydney Levy, Luisa Weiss, Rachel Cahalane, Bart Meuris, Elena Aikawa

**Affiliations:** 1 Center for Interdisciplinary Cardiovascular Sciences, Department of Medicine, Brigham and Women’s Hospital, Harvard Medical School, Boston, MA, United States; 2 School of Engineering, Bernal Institute, Health Research Institute, University of Limerick, Limerick, Ireland; 3 Department of Cardiovascular Diseases, Research Unit of Cardiac Surgery, University Hospitals Leuven, Leuven, Belgium; 4 Center for Excellence in Vascular Biology, Department of Medicine, Brigham and Women’s Hospital, Harvard Medical School, Boston, MA, United States

**Keywords:** bioprosthetic heart valve, calcification, extracellular matrix remodeling, extracellular vesicles, inflammation, macrophages

## Abstract

Bioprosthetic heart valves (BHVs) are widely used to treat aortic valve disease, and their demand is rising. However, BHV lifespan remains limited by degeneration, with patients often requiring surgical or transcatheter reintervention within a decade. BHVs lack intrinsic remodeling capabilities, and the mechanisms contributing to their failure remain poorly understood. In this review, we summarize current knowledge of BHV degeneration by integrating findings from native aortic valve disease with studies of explanted bioprosthetic valves. We discuss the structural hallmarks of degeneration, including collagen remodeling, calcification, and inflammatory cell accumulation, and examine evidence that circulating blood-derived factors infiltrate BHV tissue and contribute to degeneration progression. Particular emphasis is placed on macrophages, which are consistently identified in explanted BHVs and may promote extracellular matrix (ECM) degradation. We also highlight extracellular vesicles (EVs) as potential mediators of cardiovascular calcification and propose that macrophage-derived EVs may promote ECM remodeling beyond sites of direct cellular accumulation. In addition, we review experimental approaches, including histological imaging, *in vitro* cell culture models, and proteomic analyses, that may help define molecular pathways underlying degeneration. Together, we hypothesize a model of BHV degeneration driven by interactions among host immune cells, circulating factors, and bioprosthetic tissue. Improved understanding of these mechanisms may facilitate the development of therapeutic strategies to improve BHV durability and long-term clinical outcomes.

## 1 Introduction

Heart valve disease is a severe condition affecting 2.5% of the population (1). One of the most common and increasingly prevalent etiologies is calcific aortic valve disease (CAVD) resulting in aortic stenosis. CAVD is often associated with aging or congenital valve abnormalities, including bicuspid aortic valve. Over time, calcium progressively deposits on valvular leaflets, with hydroxyapatite crystals developing through osteogenic cell differentiation (2). The process is active, involving biomineralization and tissue remodeling, and can lead to adverse clinical outcomes, including heart failure and death (3). Despite ongoing research into disease mechanisms, there are no existing therapies or effective drug treatments to prevent or halt CAVD progression. Thus, the only current treatment options are interventional approaches, including surgical valve replacement or transcatheter valve implantation. Without valve replacement, about half of patients will die within two years following initial symptoms, making the prognosis worse than in many cancers (4).

More than 290,000 heart valve replacement procedures are performed annually, and this number is predicted to almost triple by 2050 (5). Bioprosthetic heart valves (BHVs) are widely considered the preferred option for surgical intervention, and all currently used transcatheter valves are also tissue-based prostheses. However, the long-term durability of BHVs is limited by structural valve degeneration (SVD), often requiring reintervention (6, 7). SVD is defined by intrinsic and permanent changes to the valve (e.g., calcification) and is distinct from non-structural valve dysfunction, thrombosis, and infective endocarditis (8). In a large patient cohort, degeneration was first observed at a median of 5.8 years following initial procedure (9). However, durability varies across patients and is influenced by factors including age, prosthesis type and implantation technique. Given the substantial and growing clinical burden, there is an urgent need to better understand the cellular and molecular mechanisms underlying BHV degeneration.

**Figure 1:**
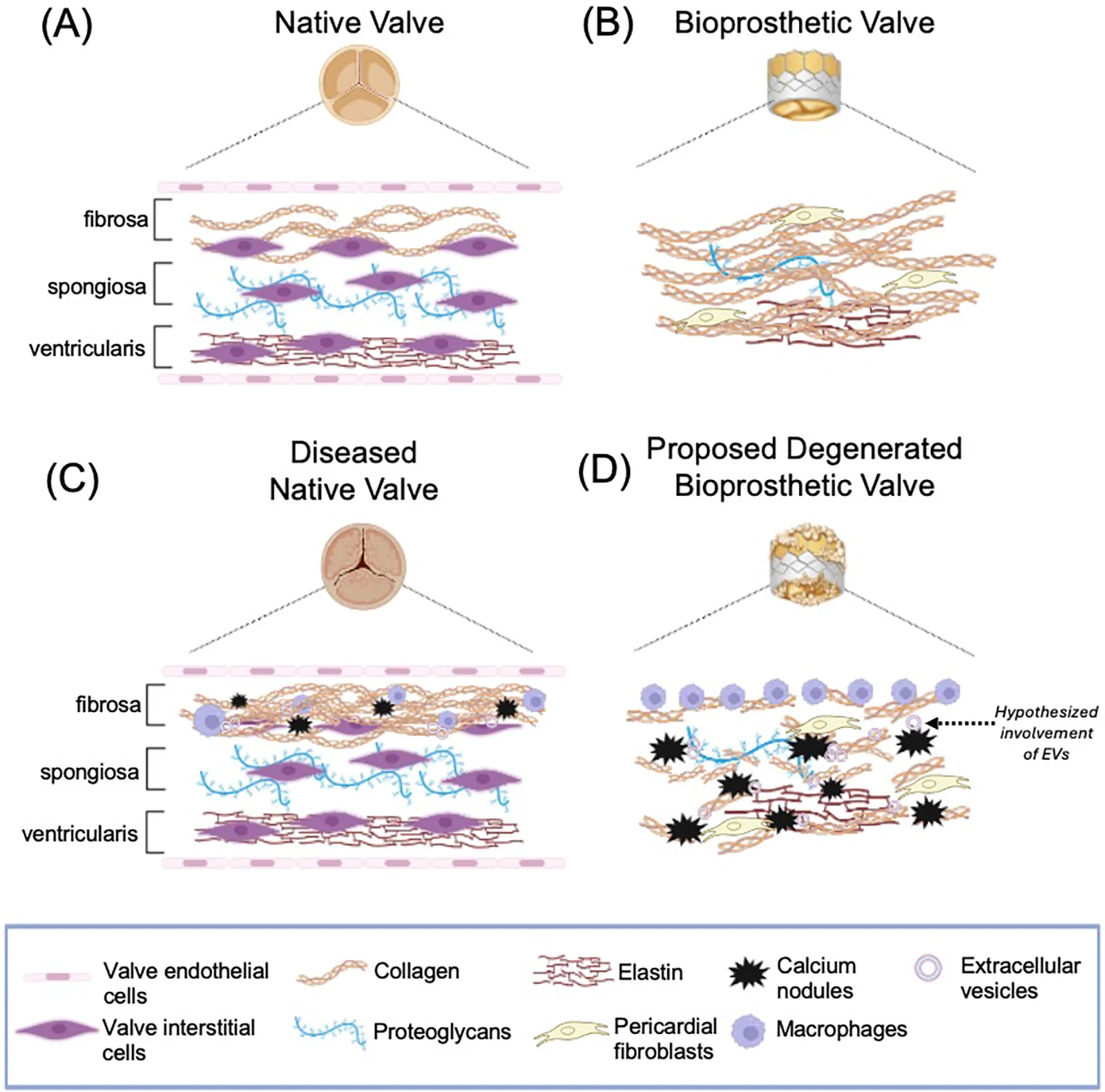
Composition of healthy and diseased native aortic valve versus healthy and degenerated bioprosthetic aortic valve. **(A)** Schematic of a healthy native aortic valve. The structure is layered, consisting of the collagen-rich fibrosa, proteoglycan-rich spongiosa, and elastin-rich ventricularis. Each layer contains VICs and the surface covered with VECs. **(B)** Schematic of a healthy bioprosthetic aortic valve. The components are similar to those of a native aortic valve, but the cells are devitalized pericardial fibroblasts. **(C)** Schematic of a diseased native aortic valve. Collagen becomes altered throughout the valve and calcium deposits within the fibrosa. Macrophages and EVs are present and promote these pathological changes. **(D)** Schematic of our proposed model of BHV degeneration. Experimentally supported processes are integrated with hypothesized pathways. The dashed arrow indicates a proposed interaction that has not been directly demonstrated in BHVs. *Created with BioRender.com.*

Insights from the more extensively characterized pathology of native aortic valve disease, particularly host-mediated and other external factors, provide context for interpreting degenerative changes in BHVs. Native valves have a highly organized extracellular matrix (ECM) composed of collagen, elastin, and proteoglycan-rich layers that contain valve interstitial cells (VICs) and are covered by valve endothelial cells (VECs) (Figure 1A) (10, 11). In contrast, BHVs are manufactured from biological tissues that are chemically fixed with glutaraldehyde before implantation, leaving the resident pericardial fibroblasts devitalized (Figure 1B) (12). As a result, BHVs lack the ability to maintain tissue integrity, synthesize matrix proteins, and repair themselves (13). Despite these biological differences, native valve disease and BHV degeneration share several pathological features. Thus, native valve disease provides a framework for investigating potential mechanisms of BHV degeneration.

This review first summarizes key features of native valve disease. It then reviews the structural pathology of degenerated BHVs, discusses current evidence regarding contributors to degeneration, and integrates these findings to propose a model of BHV degeneration.

## 2 Native aortic valve disease

Native aortic valve disease is characterized by progressive ECM remodeling that leads to leaflet thickening, stiffening, and impaired valve function. These pathological changes are driven by complex interactions among valve cells, inflammatory mediators, and surrounding tissue. A key contributor to this process is altered communication between VICs and VECs, which promotes collagen remodeling and calcium deposition (Figure 1C) (14, 15).

Collagen remodeling is a hallmark of native valve disease. Collagen undergoes crosslinking that alters tissue structure and mechanical properties. Additionally, collagen structural changes contribute to calcium phosphate nucleation, as crystals can fill in gaps between fibers (16–18). Valvular calcification progresses through initiation, propagation, and advanced stages (19). Initiation is characterized by increased presence of cells, such as macrophages, that promote inflammation and release of pro-osteogenic cytokines. In the propagation phase, microcalcification begins and mineralization via nucleation of hydroxyapatite occurs. Finally, at advanced stages, mineralization becomes more pronounced, while macrophage accumulation and inflammation decline (19, 20).

In addition to ECM and cellular changes, recent research highlights a role of extracellular vesicles (EVs) in promoting calcification (17, 20–24). These cell-released nanoparticles contribute to the pathobiology of CAVD and represent an important research target for therapeutic intervention.

Ultimately, native valve disease provides context for understanding BHV degeneration. However, BHVs lack viable VICs and VECs and the active mechanotransduction and repair mechanisms of native valves. Therefore, native disease mechanisms cannot be directly translated to BHVs. Rather, findings provide a framework for identifying potential mechanisms that can be evaluated alongside evidence directly obtained from BHVs. Together, these sources of evidence inform our proposed BHV degeneration model (Figure 1D).

## 3 Structural pathology of explanted BHVs

BHVs are manufactured from decellularized collagen-rich bovine pericardium or porcine valve tissue. Despite their initial acellular nature, explanted BHVs exhibit features of structural valve degeneration, including collagen degradation, calcification, and cell infiltration. Together, these changes contribute to progressive deterioration of valve structure and function.

Collagen is the primary component of BHV tissue, and alterations in its organization are a hallmark of degeneration. Evidence suggests collagen becomes less dense and degrades over time (25). Distinct changes also include disorganization of collagen fibers and altered crosslinking. Neotissue deposition can occur, involving the formation of new host-derived matrix on the valve (26).

Changes to collagen are linked to valve calcification. The collagen matrix supports nucleation of calcium phosphate within EVs, allowing crystals to fill in gaps between fibers and alter tissue structure (16–18). Calcific deposits contribute to leaflet stiffening, remodeling, and dysfunction.

**Figure 2:**
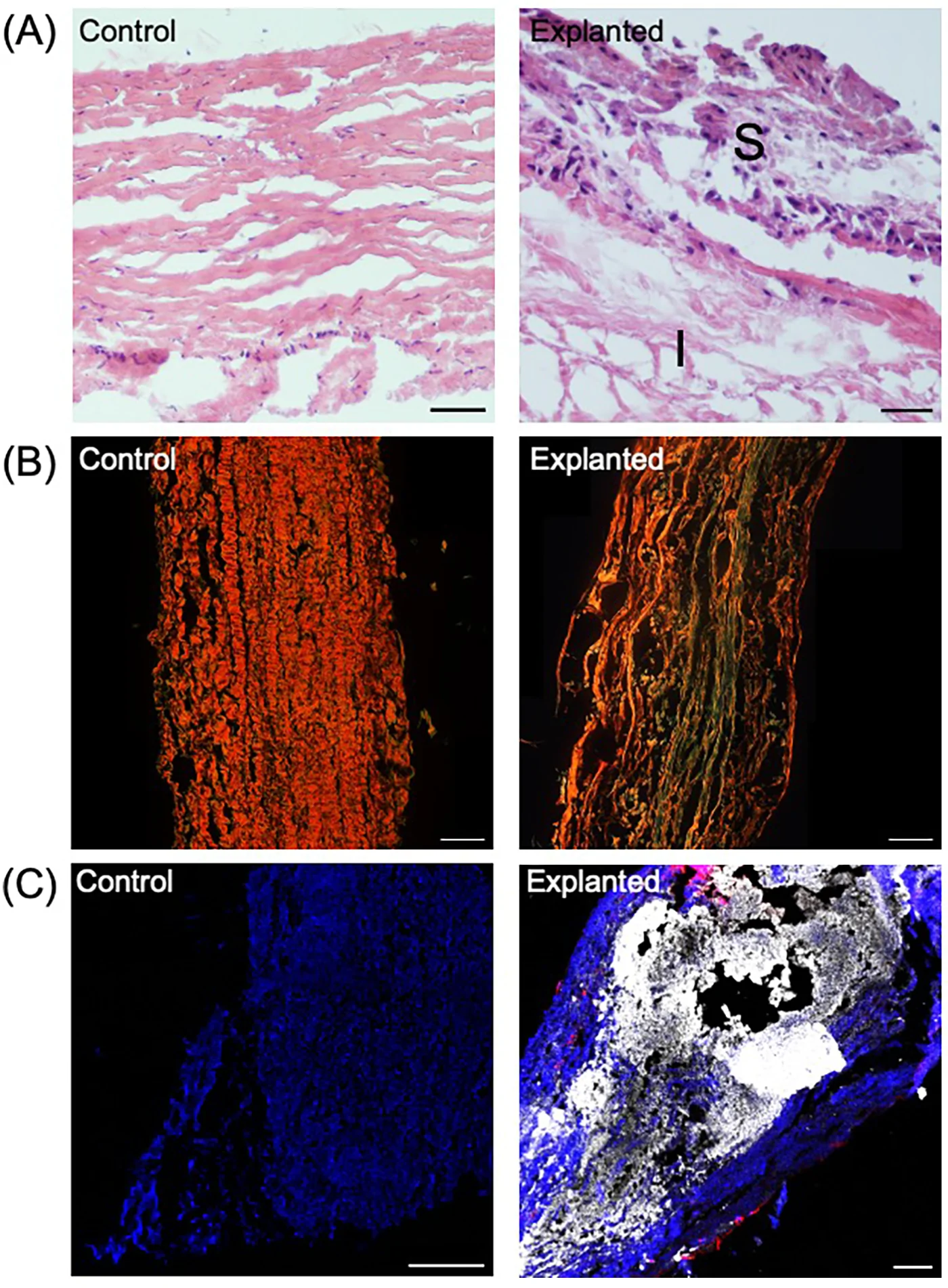
Histological features of explanted bioprosthetic heart valves. **(A)** Hematoxylin and eosin (H&E) staining of an explanted valve demonstrates host cells predominantly localize near the valve surface (S), with no cells present in the interstitium (I) compared with non-implanted control tissue. Scale bar = 50 µm. **(B)** Picrosirius Red (PSR) staining under polarized light demonstrates collagen degradation in explanted BHV tissue, characterized by increased green, thin collagen fibers compared with non-implanted control tissue. Scale bar = 100 µm. **(C)** OsteoSense demonstrates calcification deposits within explanted BHV tissue, characterized by increased white fluorescence compared with non-implanted control tissue. Vimentin immunostaining is shown in pink. Scale bar = 100 µm.

Alongside these structural changes, explanted BHVs frequently exhibit evidence of cell infiltration and ingrowth. Multiple studies have demonstrated the presence of host cells in degenerated valves, particularly under the tissue surface of the valve inflow or outflow side (27–30). Although inflammatory cell infiltration into the valve interstitium has been reported in some cases, host cells appear to predominantly localize near the tissue surface (Figure 2A). For example, Sellers et al. reported modest infiltration of CD45/leukocyte common antigen positive cells in explanted BHVs (29). While the extent of cellular infiltration into tissue remains unclear, this preferential accumulation may reflect the dense structure of bovine pericardium, which likely restricts deeper cell penetration.

Importantly, despite the localization of cells to the surface, pronounced degeneration can occur across the entire valve. Kostyunin et al. demonstrated that despite distinct increased cellular presence on human explanted porcine valves compared to bovine valves, degeneration occurred in all explants and was associated with comparable proteomic profiles (31). These findings suggest that extensive cellular presence is not a direct marker or prerequisite for advanced matrix degeneration. Thus, mechanisms beyond direct cell-ECM interactions may contribute to degeneration progression.

## 4 Degeneration drivers

### 4.1 Mechanical and material changes

The structural changes observed in BHVs likely arise from multiple interacting mechanisms, including mechanical stress and material-related factors. Valve leaflets are exposed to repetitive cyclic loading that can promote fatigue. Whelan et al. found that cyclic tensile loading of glutaraldehyde-fixed bovine pericardium samples resulted in mechanical damage that was dependent on collagen fiber alignment (32). Additionally, glutaraldehyde fixation can lead to chemical alterations that predispose tissue to degeneration. For example, exposed aldehyde groups and phospholipids can increase calcification propensity (13).

### 4.2 Circulating factors

In addition to material and mechanical factors, circulating factors may contribute to BHV degeneration. Unlike native valves, BHVs lack a protective endothelial lining and are more directly exposed to circulating blood components. Furthermore, because BHVs are acellular, proteins detected within valve tissue are likely derived from the circulation rather than produced by endogenous valve cells (6, 7, 33).

Consistent with these concepts, several plasma-derived proteins have been detected in explanted BHVs, including plasminogen, fibrinogen, and human serum albumin (31, 34, 35). Red blood cells have also been observed in explanted BHVs (6). Notably, Frasca et al. identified serum albumin and glycation products in 45 patient explants and demonstrated *in vitro* alterations in collagen alignment following exposure to these factors (34).

Lipid and oxidative modifications have also been observed in degenerated BHVs. Oxidized low-density lipoprotein has been detected in valve explants (36). Additionally, Christian et al. identified evidence of oxidative modification in BHV explants and demonstrated *in vitro* that an oxidizing environment promotes collagen degeneration (37). Valve interaction with the blood can also result in thrombosis, which may accelerate calcific processes (38).

Along with directly modifying tissue, circulating host factors may contribute to immune and inflammatory responses.

### 4.3 Immune and inflammatory response

Although BHVs are treated with glutaraldehyde to reduce immunogenicity, they remain foreign biomaterials capable of eliciting immune responses. In particular, xenoantigens such as galactose-α-1,3-galactose (*α*Gal) and N-glycolylneuraminic acid (Neu5Gc) may persist in tissue and allow for host recognition (39). Senage et al. found increased anti-αGal and anti-Neu5Gc IgGs levels six months following implantation and showed complement deposition in explants (40). Host recognition may also extend beyond carbohydrate xenoantigens as Gates et al. identified 19 tissue protein antigens associated with an adaptive immune response (41).

Importantly, these responses may promote chronic inflammation and pathogenesis. Consistent with this concept, degenerated BHV explants frequently contain immune cells, including neutrophils, multinucleated giant cells, polymorphonuclear cells, T cells, and B cells (27, 29, 31, 42). Neutrophils may contribute to degradation by releasing proteolytic enzymes including neutrophil elastase, myeloblastin/proteinase 3, and cathepsin G (31).

Of the immune cells identified in explants, macrophages are among the most prevalent. Both canonical macrophages and lipid-laden macrophages (e.g., foam cells) have been observed in degenerated BHVs (27, 31, 35, 36, 42). These cells frequently localize to regions of damage. Macrophages have been identified in areas near fragmented collagen and calcification, suggesting a relationship between macrophage accumulation and ECM remodeling (27, 30, 35). Non-lipid-laden macrophages localize to degraded ECM, and lipid-laden macrophages localize to the valve area just under the inflow or outflow side, where they may promote osteogenic inflammatory processes (27, 30).

Several mechanisms may explain how macrophages contribute to BHV degeneration. Macrophages can internalize collagen, providing a direct pathway for matrix degradation (43, 44). Additionally, they produce matrix metalloproteinases (MMPs), enzymes involved in collagen breakdown (45). MMP-9, which has been implicated in native aortic valve disease, has been identified in degenerated BHVs and localizes to areas of tissue damage, supporting its role in ECM degradation (6, 46). Furthermore, inflammatory cytokine signaling may promote macrophage-mediated osteogenic processes that result in calcification (30). Macrophages may also fuse to form foreign-body giant cells in response to material that they cannot effectively phagocytose (39).

Collectively, these findings suggest that macrophages contribute to BHV degeneration through inflammatory signaling, ECM degradation, and calcification. However, the limited penetration of these cells into the valve interstitium suggests that mechanisms beyond direct cell-ECM interactions are also involved.

### 4.4 EV-mediated remodeling and calcification

A potential mechanism by which macrophages may affect tissue regions beyond sites of direct cellular accumulation is through the release of extracellular vesicles (EVs) (47). EVs are membrane-bound nanosized particles released from cells that mediate cellular communication, transporting proteins, mRNAs, miRNAs, DNA, and lipids (48). Although the role of EVs in BHV degeneration is not fully established, evidence from other cardiovascular diseases supports their involvement in ECM remodeling and calcification (17, 20–24). EVs interact with collagen fibers and accumulate in areas of degraded collage, where they may promote microcalcification. EV-collagen interactions occur in atherosclerosis and native aortic valve calcification (17, 21, 24). In a large-scale proteomic study, Blaser et al. identified extensive EV-related protein networks in these two diseases (23).

While EVs are released by many cell types, evidence supports macrophages as an especially important source of EVs involved in cardiovascular pathology. New et al. suggested that macrophage-derived EVs containing high levels of S100A9 and Anx5 promote hydroxyapatite nucleation and microcalcification in atherosclerotic plaques (49). Furthermore, Nguyen et al. found that miRNAs carried by macrophage-released EVs may contribute to atherosclerosis (50). Although atherosclerosis is mechanistically distinct from valvular calcification, both processes involve inflammation, ECM remodeling, and pathological mineralization, making findings from atherosclerosis informative for understanding valve disease (51). In native valve disease, Xia et al. demonstrated that tsRNA-5006c carried by macrophage-derived EVs alters VIC behavior by triggering osteogenic differentiation (52). Together, these findings support a role for macrophage-derived EVs in cardiovascular tissue remodeling and calcification.

While direct evidence for EVs in BHV degeneration is limited, several characteristics of BHVs provide a rationale for investigating this mechanism. Macrophages are frequently identified in degenerated BHV explants, but they often accumulate near the surface, likely because of the dense structure of bioprosthetic tissue. Because EVs are nanosized, we hypothesize that they can penetrate deeper into the valve, reaching regions of the interstitium where matrix disruption and calcification occur. BHVs also lack the endothelial barrier present in native valves, potentially facilitating greater infiltration of circulating EVs into valve tissue. Furthermore, the degraded collagen matrix of BHVs may expose collagen voids that could provide favorable niches for EV accumulation, mineral nucleation, and calcification. Collectively, these characteristics support our hypothesis that macrophage-derived EVs may represent a mediator linking inflammation, ECM remodeling, and calcification in BHV degeneration. This has yet to be directly demonstrated in BHVs and is an important area for future work.

### 4.5 Proposed model of BHV degeneration

Based on available evidence, we propose a multifactorial model of BHV degeneration involving mechanical and material changes, circulating factors, and host immune responses (Figure 1D). Within this framework, we hypothesize that macrophage-derived EVs may link inflammation to ECM remodeling. Macrophages may contribute directly to matrix degradation through collagen internalization and MMP production while simultaneously releasing calcifying EVs. The collagen-rich matrix may provide a scaffold for EV accumulation and mineral nucleation (16–18). While their direct contribution remains to be established, EVs may represent one component of a broader multifactorial process of BHV degeneration (Table 1).

**Table 1:** Summary of proposed mechanisms contributing to bioprosthetic valve degeneration.

Proposed mechanisms	Level/type of experimental evidence	Supporting references	Remaining knowledge gaps
Mechanical fatigue and material-associated changes	Experimental studies of BHV materials	(13, 32)	Relative contribution to progressive BHV degeneration and interactions with other mechanisms
Circulating factor infiltration and tissue modification	Explant and experimental studies of BHV materials	(6, 31, 34–38, 40)	Relative contribution to progressive BHV degeneration and interactions with other mechanisms
Immune and inflammatory responses	Clinical, explant, and experimental studies in other systems	(27, 29, 31, 36, 39–42)	Relative contribution and mechanisms of specific pathways in BHV degeneration
Macrophage-mediated ECM remodeling	Explant studies of BHVs and experimental studies in other systems	(6, 27, 30, 31, 35, 36, 39, 42–46)	Direct contribution and mechanisms of macrophages in BHV ECM degradation
Macrophage-derived EV-mediated remodeling and calcification	Experimental studies primarily in atherosclerosis and native valve disease, with limited direct evidence in BHVs	(16–18, 20–24, 47, 49–52)	Direct contribution and mechanisms of macrophage-derived EVs in BHV ECM degradation

## 5 Experimental techniques

To test our model and characterize BHV degeneration, relevant approaches include histology and imaging, *in vitro* models, and proteomics.

### 5.1 Histology and imaging

Histology and imaging can characterize structural changes in BHV degeneration. Explanted bioprosthetic valve leaflets shown in Figure 2 were obtained from revision aortic valve replacement surgeries following institutional review board protocols 2021P001077 (Brigham and Women's Hospital) and S67924 (UZ Leuven) in accordance with the Declaration of Helsinki. Overall morphology can be assessed by hematoxylin and eosin (H&E) staining (Figure 2A) and changes in collagen architecture can be visualized with Picrosirius Red (PSR) staining under polarized light, where thick (mature) and thin (immature or degrading) fibers appear distinctly (53, 54). These features can indicate fibrosis, degradation, remodeling, or newly synthesized collagen (Figure 2B). Image analysis can further assess collagen organization, alignment, and crimp patterns.

Calcification can be evaluated using von Kossa staining, Alizarin Red staining, or OsteoSense imaging (55, 56). OsteoSense offers the greatest specificity and sensitivity (56, 57). The technique uses a near-infrared molecular imaging calcium tracer and can be combined with immunostaining to examine spatial relationships between calcification and cell populations (17, 55–57) (Figure 2C).

Finally, transmission electron microscopy (TEM) may identify EVs and determine their localization relative to collagen disruption or calcification.

### 5.2 *In vitro* cell culture models

Cell culture systems enable controlled investigation of mechanisms that may contribute to degeneration. THP-1 monocytes can be stimulated with phorbol 12-myristate 13-acetate (PMA) to be differentiated into macrophage-like cells (47, 58). These macrophage-like cells can be exposed to osteogenic or inflammatory conditions to investigate their mineralization potential either through direct calcification or through signaling to other cell types that deposit calcium (59, 60).

Research has incorporated calcification-permitting media with pericardium tissue to study valve disease (61). Incorporating macrophages into these models may help investigate interactions among macrophages, circulating factors, EVs, and bioprosthetic tissue.

However, THP-1 derived macrophages only partially recapitulate primary human macrophages, and static *in vitro* calcification models do not reproduce the cyclic mechanical loading experienced by valve leaflets *in vivo* (62). These limitations highlight the need for experimental systems that more closely reflect the BHV degeneration environment. Animal models may provide physiological context, but models that reproduce progressive human degeneration remain limited.

### 5.3 Proteomic approaches

Proteomics can characterize molecular pathways associated with BHV degeneration. Mass spectrometry studies of explanted BHVs have identified enrichment of complement components, neutrophil-related proteins, matrix proteases, coagulation factors, and lipid-related proteins, implicating proteolytic activity in degeneration (31). Cross-species proteomic approaches have demonstrated separation of host-formed neo-matrix from donor-matrix degeneration (63). In parallel, proteomic analyses of tissue-derived EVs from explanted aortic valves have identified disease-specific pathways associated with calcification and inflammation (23, 64). Furthermore, combined plasma and tissue proteomics have revealed sex-specific molecular signatures that may contribute to disease (10).

Integrating tissue, plasma, and EV proteomics may identify signaling pathways or networks involved in degeneration progression. Comparison of healthy and degenerative conditions may also identify novel biomarkers and therapeutic targets relevant to degeneration. However, reliable interpretation of EV-derived datasets requires careful isolation and characterization of EVs, which remain challenging (65). Standardized approaches to EV methodology will be important for applying these approaches to study BHVs.

## 6 Knowledge gaps and future directions

Despite advances in characterizing BHV degeneration, several fundamental questions remain unanswered.

The precise contribution of host immune cells in degeneration remains unclear. Future studies should investigate macrophage-derived proteins and signaling pathways while further examining contributions of neutrophils, T cells, and B cells.

Sex-specific mechanisms represent another area requiring further investigation. In native aortic valve disease, females often exhibit less calcification and greater fibrotic remodeling than males (66). While a 10-year transcatheter aortic valve study found comparable clinical degeneration between sexes (67), further research is needed to determine whether sex-specific molecular differences affect BHV degeneration.

Future studies should also directly investigate the role of EVs in BHV degeneration by characterizing their cargo, cellular origin, and spatial distribution in degenerated valves. Combining advanced imaging modalities, including electron microscopy, proteomic techniques, and *in vitro* modeling may help determine whether EVs are biomarkers of degenerative activity or active mediators of degeneration.

Addressing these questions requires more robust experimental approaches. Existing studies often rely on analyses of explanted valves, which provide only a late-stage view of degeneration progression. Future work should incorporate larger patient cohorts, matched plasma and tissue analyses, and longitudinal experiments. Given the limited availability of clinical samples, *in vitro* systems that better model the bioprosthetic environment are particularly valuable.

## 7 Conclusions and perspectives

BHVs have transformed aortic valve disease treatment, but structural degeneration remains a major limitation to their durability. Despite progress in describing BHV structural and molecular features, many mechanisms underlying their degeneration remain poorly defined. A growing body of evidence suggests matrix remodeling and degradation are active processes. Here we propose a model involving interactions among host inflammatory cells, circulating factors, and the BHV ECM. Future studies are required to precisely define these interactions.

Overall, understanding the pathways that drive BHV degeneration is essential for uncovering therapeutic strategies to extend valve durability. Determining whether macrophage-derived EVs actively contribute to tissue remodeling and calcification may represent an important step toward the development of BHVs that can better evade host-mediated degeneration. As BHV use continues to expand, particularly with widespread adoption of bioprosthetic material in transcatheter valve replacement, a deeper understanding of the mechanisms underlying structural valve degeneration is increasingly critical to improving long-term clinical outcomes.
